# Development and validation of a quality of life scale for pediatric mastocytosis

**DOI:** 10.1186/s13223-026-01051-z

**Published:** 2026-07-18

**Authors:** Aslı Berivan Topçak, Ece Tüsüz Önata, Şeyma Genç, Sefika Ilknur Kökcü Karadag, Nilay Çalışkan, Güler Yıldırım, Hamit Boloğur, Hilal Güngör, Merve Karaca Şahin, Muhammed Fatih Erbay, Hasan Tunç Şarman, Özlem Terzi, Emek Kocatürk, Frank Siebenhaar, Öner Özdemir, Deniz Özçeker

**Affiliations:** 1 Department of Pediatric Allergy and Immunology, University of Health Sciences, Prof. Dr. Cemil Tascioglu City Hospital, Istanbul, Turkey; 2 Sakarya University Faculty of Medicine, Pediatric Allergy and Immunology, Sakarya, Turkey; 3 Republic of Türkiye Ministry of Health İlkadım District Health Directorate, Samsun, Turkey; 4 Department of Public Health, Faculty of Medicine, Ondokuz Mayıs University, Samsun, Turkey; 5 Institute of Allergology, Charités – Universitätsmedizin Berlin, Corporate Member of Freie Universität Berlin and Humboldt-Universität Zu Berlin, Berlin, Germany; 6 Fraunhofer Institute for Translational Medicine and Pharmacology ITMP, Immunology and Allergology, Berlin, Germany; 7 Department of Dermatology, Bahçeşehir University School of Medicine, Istanbul, Turkey

## Abstract

**Background:**

Mastocytosis imposes a considerable burden on patients’ quality of life. While validated QoL instruments are available for adults with systemic mastocytosis, no disease-specific quality-of-life measure has yet been developed for pediatric populations.

**Objective:**

This study aimed to develop and validate two disease-specific quality of life instruments—the Pediatric Mastocytosis Quality of Life (PedMQLS) and the Pediatric Mastocytosis Quality of Life–Parent (PedParentMQLS) scales—for use in pediatric mastocytosis.

**Methods:**

A total of 51 children and their parents were recruited from two specialized tertiary centers in Turkey. Scale items were generated based on expert opinion and literature review. Content validity was evaluated using the Davis method, and construct validity was assessed through exploratory factor analysis (EFA). Internal consistency was measured using Cronbach’s alpha, and convergent validity was examined by correlating results with established dermatology-specific quality of life instruments.

**Results:**

Both scales consisted of 14 items and revealed a two-factor structure covering social and emotional domains. Pediatric Mastocytosis Quality of Life-Parent Scale showed high internal consistency (α = 0.909), while the Pediatric Mastocytosis Quality of Life Scale demonstrated acceptable reliability (α = 0.785). Significant correlations with comparable instruments supported the scales’ convergent validity.

**Conclusion:**

These newly developed scales are the first validated quality of life instruments specific for pediatric mastocytosis. This study is preliminary; validation through multicenter studies with larger sample.

**Supplementary Information:**

The online version contains supplementary material available at 10.1186/s13223-026-01051-z.

## Introduction

Mastocytosis is a rare disease characterized by clonal proliferation of mast cells in skin, bone marrow and various internal organs [1]**.** World Health Organization (WHO) classifies mastocytosis in two main categories: cutaneous mastocytosis (CM) and systemic mastocytosis (SM). The systemic form manifests mainly during adulthood and shows extracutaneous involvement, while CM develops mostly during childhood—particularly in infancy—and usually regresses spontaneously during adolescence remaining confined to skin [1–5].

In the studies conducted on adult mastocytosis, it is shown that the disease significantly affects emotional and social functions while also reducing the quality of life [6–9]. The QoL impairment in adult patients are evaluated by MC-QoL scale -a disease specific questionnaire- for adult population [10]. It has also been reported that cutaneous mastocytosis has adverse effects on quality of life in children [11, 12]. However, quality of life in pediatric mastocytosis, is usually assessed using children dermatological quality of life index (CDLQI) [11, 12]. Although the CDLQI is widely used to evaluate the impact of dermatological symptoms on daily life, it does not adequately capture disease-specific aspects of mastocytosis, including specific triggers, systemic symptoms, and parental concerns. Disease-specific quality of life instruments provide more sensitive and clinically meaningful data compared with generic scales.

This study aims to develop a disease-specific quality of life questionnaire designed to assess the impact of pediatric mastocytosis on patients’ quality of life. Accordingly, two new scales Pediatric Mastocytosis Quality of Life (PedMQLS) and Pediatric Mastocytosis Quality of Life-Parent (PedParent-MQLS) were developed that can be completed by children and their parents. These instruments offer disease specific tools to assess patients’ quality of life more accurately while allowing the individualization of treatment and psychoeducational interventions.

## Materials and methods

Children aged to 6 to 18 years—diagnosed with cutaneous mastocytosis and followed in outpatient clinics of Pediatric Allergy and Immunology in Prof. Dr. Cemil Taşcıoğlu City Hospital and Sakarya University Faculty of Medicine—along with their parents were included in this study. The diagnosis of cutaneous mastocytosis was established based on typical skin lesions, a positive Darier’s sign and absence of systemic symptoms [13]. Lesion classification included polymorphic maculopapular cutaneous mastocytosis (MPCM), monomorphic MPCM, and mastocytoma.

Exclusion criteria were age > 18 years, comorbid chronic disease, progression to systemic mastocytosis, or regular medication use. Systemic mastocytosis was ruled out by clinical and laboratory evaluation.

The demographic data, clinical characteristics, and laboratory findings of the participants were recorded along with the SCORMA (SCORing MAstocytosis) index values.

Patients were assessed with the newly developed PedMQLS and CDLQI, while parents completed the parent forms of PedParentMQLS and the Family Dermatology Life Quality Index (FDLQI). CDLQI -consisting of 10 items- was developed in 1995 to assess the impact of dermatological diseases on children aged 4 to 14 [14]. Its Turkish validation was conducted with Balcı et al. [15]. The FDLQI was developed by Basra et al. to assess the impact of pediatric dermatologic diseases on quality of life of their family members [16].

The PedMQLS was administered exclusively to children aged 6 years and older with cutaneous mastocytosis, whereas the PedParentMQLS was completed by the parent primarily responsible for the child’s daily care. The primary rationale for restricting the PedMQLS to children aged 6 years and above was that children in this age range are able to articulate their complaints more clearly and reliably, and the age threshold is consistent with that of the comparison scales used. The primary aim of the parent questionnaire is to reveal the difficulties and burden experienced by parents due to their child’s illness. The questionnaires were piloted on three children and their parents to assess comprehensibility.

The developed questionnaires are provided as Appendix A and Appendix B in the online additional file.

### Item development and expert review for content validity

During the development process of the PedMQLS and PedParentMQLS questionnaires, recommendations for designing PRO measures were taken into account [17, 18]. An expert team was assembled to identify the items assessing the symptoms of mastocytosis and their impact on quality of life.

During the item development phase, existing definitions related to mastocytosis and quality-of-life assessment, as well as previously published studies, were comprehensively reviewed. Although formal focus-group interviews were not feasible due to the rarity of pediatric mastocytosis, recurrent concerns reported by patients and parents during routine clinical follow-up, together with expert opinion and literature review, were incorporated into the questionnaire design process in accordance with patient-centered PRO development principles. As a result of this process, an initial pool of 25 items was generated for both children and parents based on the clinical and academic expertise of the research team.

All generated items were reviewed by the research team in accordance with fundamental scale development principles, including redundancy and similarity, the use of negatively worded items, the inclusion of a single judgment per item, and the use of clear and understandable language. Items with overlapping meanings were merged, inappropriate statements were removed, and the remaining items were reduced to a 19-item preliminary version for each scale, which was then submitted for content validity assessment.

The questionnaires were conceptually designed to evaluate the impact of pediatric mastocytosis on two principal domains of health-related quality of life: social/daily life functioning and emotional well-being. Although these domains guided the item generation process, no predetermined subscale structure was imposed; instead, the final two-factor structure was empirically identified through exploratory factor analysis.

Draft versions of the scales, each consisting of 19 items, separately for children and parents, were developed. Each item was assessed with a 4-point Likert-type response scale, ranging from “Never” (1 point) to “Usually” (4 points). The score is obtained by summing item responses (1–4).

Opinions of six experts including clinicians and academicians from pediatric allergy and public health were adopted to evaluate the clarity, relevance, and comprehensiveness of the draft items. Expert evaluations were assessed using Davis technique [19].

### Structural validity

Exploratory factor analysis (EFA) was used to assess the structural validity of the PedMQLS and PedParentMQLS. Prior to the analysis, the compatibility of the sample with factor analysis was evaluated using the Kaiser–Meyer–Olkin (KMO) measure of sampling adequacy and Bartlett’s test of sphericity. EFA was utilized to assess the structural validity, while Principal Axis Factoring was utilized as the extraction method and Varimax as the rotation technique. Factors with eigenvalues equal to or greater than 1 were considered appropriate factors.

### Reliability analysis

To evaluate the reliability of PedMQLS, internal consistency analyses were conducted using Cronbach’s α coefficient. Based on its results, 0.60 as the alpha coefficient was found acceptable. Using the Split-Half method, all items were divided into even and odd halves, while the internal consistency was assessed with the Guttman Split-Half Reliability Coefficient (> 0.70) and the Spearman-Brown Coefficient (> 0.70). Item analysis was conducted via item-total correlation analysis and positive item-total correlation coefficients >  + 0.25 were found acceptable.

### Development of the U.S. english version

The original Turkish versions of PedMQLS and PedParentMQLS were translated into U.S. English by two independent translators, followed by backward translation by a bilingual native speaker. The final version was approved after comparison with the original and expert review.

### Data analysis

Data analysis was performed using IBM AMOS version 23.0 and IBM SPSS version 23.0. Data distribution was assessed using the Shapiro–Wilk test and skewness–kurtosis values. Descriptive statistics were presented as frequencies, percentages, mean ± standard deviation, and minimum–maximum values.

Content validity of the scale was assessed using the Davis technique and the Content Validity Index (CVI), while structural validity was evaluated by Exploratory factor analysis (EFA). Sample adequacy for factor analysis was examined using the Kaiser–Meyer–Olkin (KMO) measure and Bartlett’s test of sphericity; Principal Axis Factoring was used as the extraction method and Varimax as the rotation technique.

Scale reliability was evaluated using item–total correlations, Cronbach’s alpha coefficient, and the split-half method. As the data were not normally distributed, the Mann–Whitney U test and Kruskal–Wallis test were used for group comparisons. Relationships between variables were analyzed using Spearman’s correlation, and p < 0.05 was considered statistically significant.

## Results

### Evaluation of demographic and clinical data

Table 1 exhibits the demographic and clinical data of 51 children diagnosed with mastocytosis and included in this study. Mean age of the participants at diagnosis was found as 12,8 ± 14,7 months and median age at diagnosis as 7 months (0–72 months) (n:26, 51% female, n = 25, 49% male) (Table 1).

**Table 1:** Demographic and clinical characteristics of children with cutaneous mastocytosis (n = 51)

Age at diagnosis (months)	Mean ± SD Median (min–max)	12,8 ± 14,7 7 (0–72)
Age at disease regression (months)	Mean ± SD Median (min–max)	53,9 ± 36,2 48 (10–138)
Current age (years)	Mean ± SD Median (min–max)	6,3 ± 3,4 6,0 (0–14)
SCORMA	Mean ± SD Median (min–max)	19,1 ± 13,2 13,6 (1,0–49,8)
Frequency variables		n (%)
Sex	Female/male	26 (51,0)/ 25 (49,0)
Accompanying allergic disease	Present	12 (23,5)
Family history of allergic disease	Present	3 (5,9)
Dermatologic symptoms	Swelling of mastocytosis lesions	35 (68,6)
	Pruritus	31 (60,8)
	Flushing	7 (13,7)
	Blistering	4 (7,8)
Gastrointestinal symptoms	Nausea	4 (7,8)
	Vomiting	4 (7,8)
	Abdominal pain	5 (9,8)
Muscle pain	Present	2 (3,9)
Triggering factors	Heat/fever	32 (62,7)
	Sun	11 (21,6)
	Food	15 (29,4)
	Stress	14 (27,5)
	Trauma	11 (21,6)
Exposure to venom	Present	10 (19,6)
Reaction to vaccination	Present	2 (3,9)
History of anaphylaxis	Present	1 (2,0)
Hepatosplenomegaly	Present	2 (3,9)
Lymphadenopathy	Present	1 (2,0)
Distribution of skin involvement	Head & neck	25 (49,0)
	Trunk	41 (80,4)
	Extremities	31 (60,8)
Type of lesions	MPCM-polymorphic	39 (86,6)
	MPCM-monomorphic	6 (13,3)
	Mastocytoma	6(13,3)
Number of lesions	1	11 (21,6)
	2–10	17 (33,3)
	11–50	9 (17,6)
	> 50	14 (27,5)
Size of lesions	< 1 cm	19 (37,3)
	≥ 1 cm	32 (62,7)
Regression type	Incomplete (decreasing number of lesions)	19 (37,3)
	Stable	30 (58,8)
	Progression (increase in number of lesions)	2 (3,9)
Recommendation/prescription for carrying an epinephrine auto-injector	Present	26(51,0)
	Absent	25(49,0)

### Results of PedMQLS and PedParentMQLS validation

#### Content validity

Davis Technique was applied to calculate CVI of PedMQLS and PedParentMQLS. In the current literature, it is emphasized that a CVI score of 0.80 and higher for each item is considered adequate [19]. According to the expert feedback, 5 items were removed and the CVI score of the remaining 14 items was calculated as 0.97.

#### Exploratory factor analysis

The construct validity of the developed PedMQLS and PedParentMQLS scales was assessed through separate EFA of the child and parent forms (Table 2). According to the results of analysis of PedMQLS, a two-factor structure with an eigenvalue higher than 1 was obtained which explains the total variance of 48.5%. Factor 1 (social life/functioning) accounted for 28.3% of variance, while factor 2 (emotions) accounted for 20.02%. The factor analysis of PedParentMQLS revealed again a two-factor structure which explains 63.05% of total variance. In the PedParentMQLS form, the social life/functioning factor accounted for 35.19% and emotions factor accounted for 27.86% of variance. The factor loading was moderate to high and exhibited a stronger structure in the parents’ form. These findings showed that the two-factor structure of both forms are supported psychometrically and that especially the PedParentMQLS form is stronger in terms of factor validity. Although items 1, 3, and 14 in the PedMQLS and item 14 in the PedParentMQLS showed negative factor loadings, they were considered clinically meaningful and were retained in the scale based on expert opinion [20, 21] (Table 2).

**Table 2:** Item statistics and exploratory factor analysis (EFA) results for PedMQLS and PedParentMQLS

Scale			Factor loadings	Item-total correlations	Eigen values	% Variance	Cumulative % Variance
Pediatric Mastocytosis quality of life (PedMQLS)							
Factor 1 (Social life/ functioning)		Item 2	0.351	0.266	3.96	28.33	28.33
		Item 3	-0.164	0.114			
		Item 4	0.210	0.524			
		Item 5	0.415	0.482			
		Item 10	0.781	0.451			
		Item 11	0.840	0.553			
		Item 12	0.796	0.694			
		Item 13	0.554	0.106			
Factor 2 (Emotions)		Item 1	-0.118	0.168	2.80	20.02	48.35
		Item 6	0.462	0.585			
		Item 7	0.733	0.268			
		Item 8	0.414	0.241			
		Item 9	0.431	0.626			
		Item 14	-0.196	0.248			
Pediatric Mastocytosis quality of life-Parent (PedParentMQLS)							
Factor 1 (Social life/ functioning)	Item 2		0.587	0.567	4.92	35.19	35.19
	Item 3		0.807	0.798			
	Item 4		0.810	0.696			
	Item 5		0.298	0.526			
	Item 10		0.460	0.560			
	Item 11		0.654	0.791			
	Item 12		0.725	0.629			
	Item 13		0.744	0.613			
Factor 2 (Emotions)	Item 1		0.351	0.624	3.90	27.86	63.05
	Item 6		0.854	0.606			
	Item 7		0.750	0.507			
	Item 8		0.699	0.667			
	Item 9		0.803	0.648			
	Item 14		-0.144	0.206			

#### The reliability of internal consistency

The internal consistency evaluates how homogeneous the total and subscale scores are within themselves, thus if they measure the same construct. Internal consistency was assessed with Cronbach’s alpha coefficient for PedMQLS and PedParentMQLS (Table 3). The overall internal consistency of 14-item scale in PedMQLS form was found as α = 0.785. At the subscale level, social life/ functioning subscale (8 items) had an alpha value of α = 0.654 while the emotions subscale (6 items) had α = 0.598. The internal consistency of PedParentMQLS was α = 0.909. In the subscale level, social life/functioning subscale had an alpha value of α = 0.875, the emotions subscale had an alpha value of α = 0.781.

**Table 3:** Internal consistency of the Pediatric Mastocytosis Quality of Life scales (child and parent versions)

Subscale	Items	Cronbach’s α	
		Pediatric Mastocytosis Quality of Life PedMQLS	Pediatric Mastocytosis Quality of Life-Parent (PedParentMQLS)
Social life/ functioning	8 items	0.654	0.875
Emotions	6 items	0.598	0.781
Total scale	14 items	0.785	0.909

**Fig. 1:**
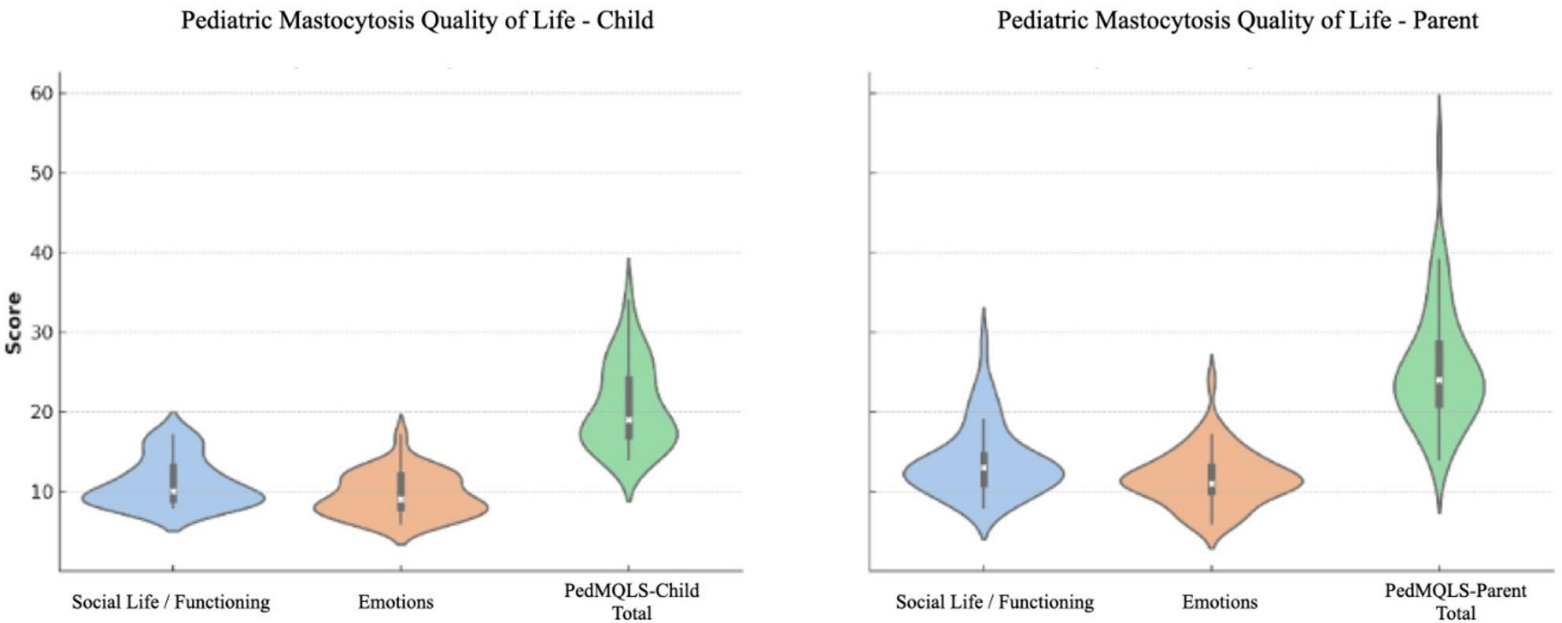
Distribution of PedMQLS (child) and PedParentMQLS (parent) scores. Panel displays subscale (Social life/functioning; Emotions) and Total scores as summary distributions (e.g., box-and-whisker plots). Higher scores reflect greater impairment in health-related quality of life

The distributions of the quality-of-life scale scores for children with mastocytosis and their parents are presented as Fig. 1.

#### Evaluation of scale scores and associated factors

The mean of total scores of PedMQLS was calculated as 20,6 ± 5,2 and the median as 19 (14–34). The mean score of the PedParentMQLS scale was 25,4 ± 7,4 while the median was 24 (14–53). In addition to the subscale scores of both scales, the scores of the CDLQI and the FDLQI administered in the study are presented as Table 4.

**Table 4:** Distribution of quality-of-life scores for children with mastocytosis and their parents

Scale scores	N	Mean ± SD	Median (min–max)
Social life scale of children	33	11,1 ± 3,0	10 (8–17)
Emotional scale of children	33	9,5 ± 2,7	9 (6–17)
Total scale of children	33	20,6 ± 5,2	19 (14–34)
Social life scale of parents	51	13,7 ± 4,4	13 (8–29)
Emotional scale of parents	51	11,7 ± 3,5	11 (6–24)
Total scale of parents	51	25,4 ± 7,4	24 (14–53)
Children’s Dermatology Life Quality Index (CDLQI)	33	3,1 ± 3,2	3 (0–14)
Parent Dermatology Life Quality Index (PDLQI)	51	15,8 ± 11,2	13 0–56)

According to the conducted analyses, PedMQLS scale scores demonstrated significant differences in terms of some clinical characteristics (Table 5). The social life subscale scores of PedMQLS revealed significant difference in relation to presence of flushing (p = 0.030), trauma as a triggering factor (p = 0.015), venom exposure (p = 0.037), and SCORMA scores (< 20/ ≥ 20) (p = 0,010). Additionally, there was a nearly significant difference in terms of age at diagnosis (0–12 months/ > 12 months) and presence of gastrointestinal (GI) symptoms (p = 0.067 and p = 0.093, respectively). Similarly, emotional scale scores of PedMQLS showed significant difference with flushing (p = 0.020), GI symptoms (p = 0.026), and SCORMA scores (p = 0.013), as well as with the regression types of the disease (p = 0.015). In terms of total scale of PedMQLS, there was a significant difference with flushing (p = 0.021), GI symptoms (p = 0.031), trauma (p = 0.042), venom exposure (p = 0.045), and SCORMA score (p = 0.005) (Table 5).

**Table 5:** Comparison of PedMQLS and CDLQI scores by demographic and clinical characteristics (child sample)

Variables		PedMQLS social life	PedMQLS emotional scale	PedMQLS total	CDLQI
Age at diagnosis	0–12 months / > 12 months	0,067	0,491	0,166	0,210
Current age (years)	6–11 / ≥ 12	0,502	0,164	0,319	0,737
Sex	Female/male	0,104	0,561	0,255	0,305
Accompanying atopic disease	Absent /present	0,790	0,289	0,700	0,681
Family history of allergic disease	Absent /present	0,260	0,382	0,262	0,453
Dermatologic symptoms	Swelling of mastocytosis lesions: absent/present	0,657	0,278	0,488	0,553
	Pruritus: absent/present	0,273	0,536	0,406	0,583
	Flushing: absent/present	**0,030**	**0,020**	**0,021**	0,202
GI symptoms	Absent/present	0,093	**0,026**	**0,031**	**0,023**
Triggering factors	Heat-fever: absent/present	0,545	0,454	0,502	0,755
	Sun: absent/present	0,240	0,933	0,361	0,355
	Food: absent/present	0,221	0,919	0,420	0,557
	Stress: absent/present	0,406	0,138	0,214	0,159
	Trauma: absent/present	**0,015**	0,212	**0,042**	**0,033**
Exposure to venom	Absent / Present	**0,037**	0,109	**0,045**	0,210
Shape of lesions (MPCM)	Polymorphic/ monomorphic	0,298	0,890	0,522	0,579
Number of lesions	1–50/ > 50	0,254	0,087	0,133	0,077
Size of lesions	< 1 cm/ ≥ 1 cm	0,296	0,306	0,318	0,107
Regression type	Incomplete/stable/worsening	0,183	**0,015**	0,083	**0,036**
SCORMA score	< 20 (below mean)/ ≥ 20 (above mean)	**0,010**	**0,013**	**0,005**	**0,005**

Table 6 presents the comparison of parents’ scale scores according to their demographic and clinical characteristics. The social life scale scores of PedParentMQLS revealed significant differences in relation to current age of children (p = 0.038), presence of swelling of mastocytosis lesions (p = 0.005), presence of pruritus (p = 0.025), presence of flushing (p = 0.007), GI symptoms (p = 0.034), heat-fever as a triggering factor (p = 0.009), presence of trauma (p = 0.002), number of lesions (1–50/ > 50) (p = 0,003), regression type (p = 0.038) and SCORMA scores (< 20/ ≥ 20) (p = 0,001).

**Table 6:** Comparison of PedParentMQLS and PDLQI scores by demographic and clinical characteristics (parent sample)

Variables		PedMQLS Social life	PedMQLS emotional scale	PedMQLS total	CDLQI
Age at diagnosis	0–12 months / > 12 months	0,067	0,491	0,166	0,210
Current age (years)	6–11 / ≥ 12	0,502	0,164	0,319	0,737
Sex	Female/male	0,104	0,561	0,255	0,305
Accompanying atopic disease	Absent /present	0,790	0,289	0,700	0,681
Family history of allergic disease	Absent /present	0,260	0,382	0,262	0,453
Dermatologic symptoms	Swelling of mastocytosis lesions: absent/present	0,657	0,278	0,488	0,553
	Pruritus: absent/present	0,273	0,536	0,406	0,583
	Flushing: absent/present	**0,030**	**0,020**	**0,021**	0,202
GI symptoms	Absent/present	0,093	**0,026**	**0,031**	**0,023**
Triggering factors	Heat-fever: absent/present	0,545	0,454	0,502	0,755
	Sun: absent/present	0,240	0,933	0,361	0,355
	Food: absent/present	0,221	0,919	0,420	0,557
	Stress: absent/present	0,406	0,138	0,214	0,159
	Trauma: absent/present	**0,015**	0,212	**0,042**	**0,033**
Exposure to venom	absent/present	**0,037**	0,109	**0,045**	0,210
Shape of lesions (MPCM)	Polymorphic/ monomorphic	0,298	0,890	0,522	0,579
Number of lesions	1–50/ > 50	0,254	0,087	0,133	0,077
Size of lesions	< 1 cm/ ≥ 1 cm	0,296	0,306	0,318	0,107
Regression type	Incomplete/stable/worsening	0,183	**0,015**	0,083	**0,036**
SCORMA score	< 20 (below mean)/ ≥ 20 (above mean)	**0,010**	**0,013**	**0,005**	**0,005**

The emotional scale scores of PedParentMQLS revealed significant differences in relation to current age of children (p = 0.008), presence of swelling of mastocytosis lesions (p = 0.007), flushing (p = 0.036), heat-fever as a triggering factor (p = 0.0148), presence of trauma (p = 0.006), number of lesions (p = 0,006), regression type (p = 0.003) and SCORMA scores (p < 0,001).

The total scale scores of PedParentMQLS revealed significant difference in relation to current age of children (p = 0.012), presence of swelling of mastocytosis lesions (p = 0.005), presence of pruritus (p = 0.044), presence of flushing (p = 0.009), GI symptoms (p = 0.043), heat-fever as a triggering factor (p = 0.049), presence of trauma (p = 0.003), number of lesions (p = 0,003), regression type (p = 0.015) and SCORMA scores (p < 0,001).

The scores of the FDLQI were found to differ significantly in terms of age of children at diagnosis (0–12 months/ > 12 months) (p = 0,017), current age of children (p < 0.001), presence of swelling of mastocytosis lesions (p < 0.001), GI symptoms (p = 0.048), heat-fever as a triggering factor (p = 0.003), food as a triggering factor (p = 0.001), presence of trauma (p = 0.015), number of lesions (p = 0,004), regression type (p = 0.023) and SCORMA scores (p = 0,002). (Table 6).

#### Known-groups validity

Convergent validity is conducted to assess whether PedMQLs and PedParentMQLS truly measures the impairment in quality of life. Therefore, Pearson correlation coefficients were calculated between total and subscale scores of the PedMQLS and those of other quality of life scales. CDLQI and FDLQI were the other instruments used as anchors for comparison (Table 7).

**Table 7:** Correlations among all quality-of-life scales, clinical variables, and laboratory parameters

Scales	1	2	3	4	5	6	7	8
	r*; p	r; p	r; p	r; p	r; p	r; p	r; p	r; p
CDLQI	1,0	0,14; 0,434	0,80; ** < 0,001**	0,71; ** < 0,001**	0,85; ** < 0,001**	0,15; 0,413	0,42; **0,014**	0,32 0,071
FDLQI		1,0	0,27; 0,129	0,36; **0,042**	0,31; 0,071	0,75; ** < 0,001**	0,66; ** < 0,001**	0,75; ** < 0,001**
PedMQLS Social life scale			1,0	0,69; ** < 0,001**	0,93; ** < 0,001**	0,34; **0,049**	0,44; **0,011**	0,44; **0,010**
PedMQLS Emotional scale				1,0	0,90; ** < 0,001**	0,29; 0,105	0,56; **0,001**	0,46; **0,008**
PedMQLS Total scale					1,0	0,34; 0,052	0,54; **0,001**	0,49; **0,004**
PedParentMQLS social life scale						1,0	0,68; ** < 0,001**	0,90; ** < 0,001**
PedParentMQLS emotional scale							1,0	,92; ** < 0,001**
PedParentMQLS total scale								1,0
Alkaline phosphatase	-0,11; 0,53	-0,22; 0,157	0,01; 0,985	-0,31; 0,092	-0,14; 0,466	-0,31; **0,042**	-0,12; 0,444	-0,22; 0,152
Lactate dehydrogenase	-0,28; 0,130	-0,07; 0,662	-0,13; 0,489	-0,42; **0,020**	-0,31; 0,097	0,03; 0,829	-0,03; 0,859	0,01; 0,958
Tryptase	-0,37; **0,039**	0,21; 0,137	-0,15; 0,415	-0,21; 0,276	-0,24; 0,203	0,24; 0,101	-0,02; 0,888	0,13; 0,365
Vitamin D	-0,26; 0,147	-0,04; 0,806	-0,32; 0,076	-0,36; **0,045**	-0,37; **0,038**	-0,04; 0,803	-0,06; 0,706	-0,05; 0,741
Number of lesions	0,29; 0,108	0,45; **0,001**	0,25; 0,163	0,31; 0,082	0,30; 0,091	0,48; ** < 0,001**	0,44; **0,001**	0,49; ** < 0,001**

^*^r: correlation coefficient, 1- CDLQI, 2- FDLQI, 3- PedMQLS social life scale, 4- PedMQLS emotional scale, 5- PedMQLS total scale, 6- PedParentMQLS social life scale, 7 PedParentMQLS emotional scale, 8- PedParentMQLS total scale

There was a statistically significant positive and linear relationship between the CLDQI and the following scales: FDLQI (r = 0,14; p = 0,434), PedMQLS social life scale (r = 0,80; p < 0,001), PedMQLS emotional scale (r = 0,71; p < 0,001), PedMQLS total scale (r = 0,85; p < 0,001), PedParentMQLS social life scale (r = 0,15; p = 0,413), PedParentMQLS emotional scale (r = 0,42; p = 0,014), and PedParentMQLS total scale (r = 0,32; p = 0,071).

The FDLQI showed significant and strong correlations with the following scales: PedParentMQLS social life scale (r = 0,75; p < 0,001), PedParentMQLS emotional scale (r = 0,66; p < 0,001), and PedParentMQLS total scale (r = 0,75; p < 0,001).

There was a significant relationship between PedMQLS social life scale and the following scales: PedMQLS emotional scale (r = 0,69; p < 0,001), PedMQLS total scale (r = 0,93; p < 0,001), PedParentMQLS social life scale (r = 0,34; p = 0,049), PedParentMQLS emotional scale (r = 0,44; p = 0,011), and PedParentMQLS total scale (r = 0,44; p = 0,010).

There was a significant correlation between PedMQLS emotional scale and the following scales: PedMQLS total scale (r = 0,90; p < 0,001), PedParentMQLS emotional scale (r = 0,56; p = 0,001), and PedParentMQLS total scale (r = 0,46; p = 0,008).

PedMQLS total scale showed significant correlations with following scales: PedParentMQLS social life scale (r = 0,34; p = 0,052), PedParentMQLS emotional scale (r = 0,54; p = 0,001) and PedParentMQLS total scale (r = 0,49; p = 0,004).

There were significant and strong correlations between PedParentMQLS social life scale and following scales: PedParentMQLS emotional scale (r = 0,68; p < 0,001), PedParentMQLS total scale (r = 0,90; p < 0,001). PedParentMQLS emotional and total scales also showed significant and strong correlation (r = 0,92; p < 0,001).

Additionally, the number of lesions had significant relationship with following scales: FDLQI (r = 0,45; p = 0,001), PedParentMQLS social life scale (r = 0,48; p < 0,001), PedParentMQLS emotional scale (r = 0,44; p = 0,001) and PedParentMQLS total scale (r = 0,49; p < 0,001).

Significant correlations between PedMQLS and PedParentMQLS scores indicated consistency between child- and parent-reported quality-of-life impairment.

Tryptase and other biochemical parameters were within the reference ranges and generally showed no significant correlation with the scale scores. However, significant negative correlations were found between LDH levels and PedMQLS emotional scale (r = -0,42; p = 0,020), between tryptase levels and CDLQI (r = -0,37; p = 0,039), between vitamin D levels and PedMQLS emotional (r = -0,36; p = 0,045) and total scales (r = -0,37; p = 0,038).

## Discussion

This study developed two new disease-specific quality of life scales—PedMQLS for children aged 6–18 years and PedParentMQLS for parents of pediatric mastocytosis patients aged 0–18 years—and demonstrated their validity and reliability in assessing the impact of the disease on quality of life, including its psychosocial dimensions.

A wide spectrum of symptoms are reported associated with mastocytosis [22]. In our patient group, frequencies of swelling of mastocytosis lesions (68%), pruritus (60%), and blistering (7%) are in line with previous literature [2, 3, 23–27]. These findings indicate that the clinical characteristics of the patient group in our study reflect the typical clinical presentation and course of childhood mastocytosis.

The PedMQLS and PedParentMQLS consist of 14 items, comprising a total score and two subdomains: emotions and social life/functioning. While the parent questionnaire demonstrated high internal consistency across all domains, the child questionnaire showed lower but still acceptable consistency due to the limited sample size. These scales allow the assessment of quality-of-life impairment in pediatric mastocytosis through both the total score and subdomain scores. Higher scores indicate greater impairment.

In the evaluation of scale scores, results of PedMQLS and PedParentMQLS indicate that the greatest impact is on the social life/ functioning domain, both for pediatric and parent population. However, the small differences between the mean scores of the two subdomains (1 and 2 points) show that the impact of mastocytosis across different dimensions of health-related quality of life (HRQoL) is uniform in general.

The convergent validities of PedMQLS and PedParentMQLS were found to be good. PedMQLS and PedParentMQLS scores showed strong and significant correlation with CDLQI and FDLQI scores which are well established and valid comparison tools. This supports the idea that these scales measure the constructs they intend to assess. Additionally, when factors associated with scale scores are examined, the PedMQLS and PedParentMQLS were found to be related to certain variables missed by CDLQI and FDLQI. Thus, the newly developed PedMQLS and PedParentMQLS are more comprehensive.

While the number of lesions does not affect the quality of life in children, it has a significant impact on parents’ quality of life, particularly in the domains of social functioning, emotional well-being, and overall scores. Both cutaneous and gastrointestinal (GI) symptoms, along with the SCORMA index, were found to influence the quality of life of both children and their parents. Moreover, trauma has been identified as a major trigger associated with a deterioration in quality of life.

In the correlation analysis, the following are defined as important factors affecting total scores of PedMQLS and PedParentMQLS: flushing, GI symptoms, trauma as a triggering factor, exposure to venom and SCORMA. Age at diagnosis affects scores of the social domain, while regression type affects scores of the emotional domain. In pediatric mastocytosis, 91% of patients have dermatological symptoms while 20% of patients have accompanying GI symptoms, therefore quality of life assessment is important [28].

Although serum tryptase, LDH, ALP, and vitamin D levels were within age-adjusted reference ranges in all patients, negative correlations with quality-of-life scores were observed. Since no clinically abnormal biochemical values were detected, these findings should be interpreted as exploratory statistical associations rather than evidence of clinically significant biochemical abnormalities. These associations may reflect subtle inter-individual variability or the effect of the limited sample size and require confirmation in larger cohorts.

### Strengths and limitations

This study has several limitations. First, the PedMQLS and PedParentMQLS questionnaires are not applicable to cases of systemic mastocytosis or hereditary alpha-tryptasemia, as these instruments were specifically developed for pediatric patients with cutaneous mastocytosis. However, since cutaneous mastocytosis represents the most common form of mastocytosis in the pediatric population, the clinical applicability of these questionnaires remains high. In addition, due to the limited cohort size, comparisons across different age groups (6–10, 11–15, and 16–18 years) could not be performed. Furthermore, the inclusion of only patients with cutaneous mastocytosis and the frequent spontaneous regression of this disease during adolescence may limit the practical applicability of the developed scale to a narrower age range. Both child-reported and parent-reported questionnaires were administered to the same cohort not only to validate the instruments but also to explore potential differences between children’s self-reported quality of life and parental perceptions. However, the limited sample size may have restricted the ability to fully elucidate these differences. Moreover, the cross-sectional design of the study limits the ability to evaluate factors affecting quality of life over time. As the present study represents a preliminary cross-sectional validation study with a limited sample size, MCID analysis was not performed. Future multicenter longitudinal studies are planned to evaluate responsiveness and clinically meaningful change thresholds of the scales.

The major strength of this study lies in being the first to investigate quality of life and its determinants in pediatric patients with mastocytosis. This study serves as a preliminary investigation, and a multicenter study involving a larger patient population is planned in the future to validate these findings.

## Conclusions

To conclude, PedMQLS and PedParentMQLS are the first valid and reliable disease-specific quality-of-life assessment tools developed for pediatric mastocytosis. By encompassing emotional and social functioning, these scales enable evaluation of quality of life in both children and their parents. These instruments provide important clinical information. Further multicenter studies with larger sample sizes are planned to enhance generalizability.

## Supplementary Information

Supplementary material 1.

## Data Availability

The data supporting the findings of this study are available from the corresponding author upon reasonable request.

## Abbreviations

ALP: Alkaline phosphatase
CDLQI: Children’s dermatology life quality index
CVI: Content validity index
CM: Cutaneous mastocytosis
EFA: Exploratory factor analysis
FDLQI: Family dermatology life quality index
KMO: Kaiser–Meyer–Olkin measure
LDH: Lactate dehydrogenase
MPCM: Maculopapular cutaneous mastocytosis
PedMQLS: Pediatric Mastocytosis Quality of Life
PedParentMQLS: Pediatric Mastocytosis Quality of Life-Parent
SCORMA: SCORing MAstocytosis
SPSS: Statistical package for social sciences
SM: Systemic mastocytosis
WHO: World Health Organization
