# Supplementary material for: Development and validation of a quality of life scale for pediatric mastocytosis
Source: Allergy Asthma Clin Immunol. 2026 Jul 18;22:43. doi: 10.1186/s13223-026-01051-z (PMC13386695; doi:10.1186/s13223-026-01051-z)
Supplement: Supplementary file 1 — Supplementary material 1. [file 13223_2026_1051_MOESM1_ESM.docx]

**Appendix A. Pediatric Mastocytosis Quality of Life Questionnaire**

This questionnaire has been designed to help us better understand the difficulties faced by children with mastocytosis. Using the scale below, please answer how often difficulty you have with each of the issues described below during the past month by selecting the appropriate option.

|  | **NEVER** | **SOMETIMES** | **OFTEN** | **USUALLY** |
| --- | --- | --- | --- | --- |
| I am suffering from itching, redness, and swelling attacks on my skin. |  |  |  |  |
| I experience difficulty or anxiety when choosing food or drinks due to my illness. |  |  |  |  |
| I refrain from going to parks, gardens, or rural areas due to fear of being stung by bees or wasps. |  |  |  |  |
| I am anxious about participating in exercise, running and other sports activities. |  |  |  |  |
| I have difficulty sleeping/ I cannot sleep well/ I think I cannot get enough sleep due to my illness. |  |  |  |  |
| I am afraid of having an anaphylactic shock due to my illness. |  |  |  |  |
| I am afraid that if I need my adrenaline medicine because of my illness, I might not be able to use it. |  |  |  |  |
| I feel unhappy or sad because of my illness. |  |  |  |  |
| I have concerns about my future due to my illness. |  |  |  |  |
| My school life is negatively affected by my illness. |  |  |  |  |
| Due to my illness, I am unable to spare time to meet with friends or relatives. |  |  |  |  |
| I think my illness has a negative impact on my family's financial situation. |  |  |  |  |
| I don’t like when people ask me about my illness, so I don’t want to meet new people or go to new places. |  |  |  |  |
| I feel distressed when asked about my skin lesions and illness. |  |  |  |  |

**Appendix B. Pediatric Mastocytosis Quality of Life Questionnaire – Parent.**

This questionnaire has been designed to help us better understand the difficulties faced by parents of children with mastocytosis. Using the scale below, please answer how often difficulty you have with each of the issues described below during the past month by selecting the appropriate option.

|  | **NEVER** | **SOMETIMES** | **OFTEN** | **USUALLY** |
| --- | --- | --- | --- | --- |
| I am having trouble with cutaneous pruritus, erythema and swelling attacks on my child's skin. |  |  |  |  |
| I experience difficulty or anxiety when choosing food or drinks due to my child's illness. |  |  |  |  |
| I refrain from taking my child to parks, gardens, or rural areas due to fear of my child being stung by bees. |  |  |  |  |
| I am anxious about my child's participation in exercise, running and other sports activities. |  |  |  |  |
| I have difficulty sleeping/ I cannot sleep well/ I think I cannot get enough sleep due to my child's illness. |  |  |  |  |
| I am afraid that my child may have an anaphylactic shock. |  |  |  |  |
| I am afraid that, if my child needs adrenaline treatment due to his/her illness, I might not be able to administer it. |  |  |  |  |
| I feel unhappy or sad because of my child's illness. |  |  |  |  |
| I have concerns that my child's illness will have a negative impact on his/her future work/family/education/social life. |  |  |  |  |
| My work life is negatively affected by my child's illness. |  |  |  |  |
| Due to my child's illness, I am unable to spare time to meet with friends or relatives. |  |  |  |  |
| My child's illness has a negative impact on our family's financial situation. |  |  |  |  |
| I do not want to meet new people or go to new places because I feel uncomfortable being asked questions about my child's illness. |  |  |  |  |
| I feel distressed when asked about my child's skin lesions and illness. |  |  |  |  |
